# Fragmentation of plastic objects in a laboratory seawater microcosm

**DOI:** 10.1038/s41598-020-67927-1

**Published:** 2020-07-02

**Authors:** Jan Gerritse, Heather A. Leslie, Caroline A. de Tender, Lisa I. Devriese, A. Dick Vethaak

**Affiliations:** 1 Deltares, Unit Subsurface and Groundwater Systems, Daltonlaan 600, 3584 BK Utrecht, The Netherlands; 2 Department of Environment and Health, Vrije Universiteit Amsterdam, De Boelelaan 1085, 1081 HV Amsterdam, The Netherlands; 3 Department of Applied Mathematics, Computer Science and Statistics, Ghent University, Krijgslaan 281 S9, 9000 Ghent, Belgium; 4 Plant Sciences Unit, Flanders Research Institute for Agriculture, Fisheries and Food (ILVO), Burgemeester Van Gansberghelaan 92, 9820 Merelbeke, Belgium; 5 Flanders Marine Institute (VLIZ), InnovOcean Site, Wandelaarkaai 7, 8400 Ostend, Belgium; 6 Deltares, Unit Marine and Coastal Systems, Boussinesqweg 1, 2629 HV Delft, The Netherlands

**Keywords:** Microbiology, Molecular biology, Environmental sciences, Ocean sciences, Materials science

## Abstract

We studied the fragmentation of conventional thermoplastic and compostable plastic items in a laboratory seawater microcosm. In the microcosm, polyurethane foams, cellulose acetate cigarette filters, and compostable polyester and polylactic acid items readily sank, whereas polyethylene air pouches, latex balloons, polystyrene foams and polypropylene cups remained afloat. Microbial biofilms dominated by Cyanobacteria, Proteobacteria, Planctomycetes and Bacteriodetes grew on the plastics, and caused some of the polyethylene items to sink to the bottom. Electrical resistances (ER) of plastic items decreased as function of time, an indication that seawater had penetrated into microscopic crevices in the plastic that had developed over time. Rate constants for ER decrease in polyethylene items in the microcosm were similar to tensile elongation decrease of polyethylene sheets floating in sea, measured previously by others. Weight loss of plastic items was ≤ 1% per year for polyethylene, polystyrene and polypropylene, 3–5% for latex, polyethylene terephthalate and polyurethane, 15% for cellulose acetate, and 7–27% for polyester and polylactic acid compostable bags. The formation of microplastics observed in the microcosm was responsible for at least part of the weight loss. This study emphasizes the need to obtain experimental data on plastic litter degradation under conditions that are realistic for marine environments.

## Introduction

Millions of tonnes of plastic waste are estimated to enter the oceans annually^1,2^. The issue of widespread plastic waste in the environment is exacerbated by the durability and persistence of these materials in the environment^3–17^. In the marine environment plastic breaks up into smaller particles^18–20^. An estimated 13% to 32% of the total weight of buoyant plastics in the oceans consists of microplastic particles of 0.3–5 mm in size^14,21,22^. It is currently unknown how and at which rates fragmentation of plastic proceeds. We also do not know to which degree biodegradation contributes to the mineralization of plastic in seawater^23–32^. This lack of data limits our capability to assess and predict the fate and residence times of plastic litter in marine ecosystems. The present study sought to shed some light on plastic litter fate in a marine microcosm to learn more about the processes and rates that could be observed.

Fragmentation of plastics is thought to be initiated by polymer chain backbone weathering through exposure to sunlight (UV), oxidants, hydrolysis and physical shearing, for example through currents, waves, or friction with sand^4,33–40^. The oxidation and shortening of polymer chains and leaching of plasticizers makes plastic materials brittle and stimulates the formation of surface cracks and fragmentation^18,19^. As a result micro- and nanometer sized plastic particles may be released from the surface of larger fragments^19^. In time this can result in the generation of numerous micro- and nanoplastic particles from a single plastic object^18^. In theory, one bag composed of two plastic sheets 50 cm × 40 cm × 50 µm thick could generate 20 particles with a volume of 1 mm^3^, 20 million particles with a volume of 1 µm^3^ or 20 trillion particles with a volume of 1 nm^3^.

The size of the plastic particles is important because it affects their potential hazard to individual organisms, communities, and ecosystems. Larger plastic litter items may be eaten by or cause entanglement of marine fish, birds and mammals, while the micro- and nanoplastic particles are more prone to being ingested not only by large, but also by smaller invertebrates such as mussels and zooplankton with the potential for accumulation in food chains^19,41^.

Fragmentation also affects plastic litter transport through marine systems because smaller particles are transported differently horizontally and vertically than larger items^42–48^. Smaller particles have a relatively large exposed surface area compared to their volume. This may result in increased degradation rates, adsorption sites per unit mass and reduced buoyancy (upon biofouling), resulting in transfer of microplastic particles from the sea surface to the water column or sediment^9,11,14,18,48^. The larger specific surface area generated through fragmentation increases contact with water with faster leaching or sorption rates for chemicals and additional area for biofouling^49^.

In 2018, about 359 million tonnes plastic were produced globally, of which 62 million tonnes in Europe. About 80% consisted of thermoplastics with polymer backbones of polyethylene (PE), polypropylene (PP), polyvinylchloride (PVC), polyurethane (PU), polystyrene (PS) or polyethylene terephthalate (PET)^50^. The material composition, e.g. chain backbone, crosslinking and additives, affects to a high degree the repertoire of mechanisms and rates of abiotic and biological degradation that can occur. Polymers with a carbon–carbon backbone, high molecular weight, and few functional groups, such as PE, PP, PS and PVC, are very resistant to degradation^9^. Ultraviolet (UV) light from the sun produces breaks in for example PE, PP, PS and PVC polymer chains. But in marine ecosystems such plastic particles are readily transferred downwards and often become buried in the sediment. Floating plastic particles become rapidly covered by biofilms, which protect them from UV radiation^51,52^ and weigh them down, causing them to sink. The timescale of the mineralization process of most plastic materials with a carbon–carbon backbone in the marine environment is usually estimated at decades or longer^9,24,33,38^.

Plastic materials with heteroatoms in the main polymer chain are susceptible to hydrolysis^9^. In the marine environment, cleavage of the ester bonds in PET and PU and amide bonds in nylon can occur through abiotic hydrolysis, photolysis and oxidation. In addition, biodegradation of PET and PU may be significant, since microorganisms that are capable of this process can readily be isolated from the environment, including marine systems^26,27,30,53,54^.

Microorganisms in biofilms are sometimes able to catalyze the partial or complete mineralization of plastic to energy, biomass and inorganic molecules such as carbon dioxide, water, and/or methane, hydrogen and ammonia^21–30,53–61^. The biodegradability of plastic materials is usually determined under conditions optimized for high metabolic rate (temperature, nutrients, pH) such as in sewage sludge, landfill, soil or compost, but not under conditions which prevail in marine environments^57^. Therefore, such tests are unreliable indicators of the fate of plastic items in the sea.

One way proposed to reduce the persistence of plastic objects in the environment is to use polymers that mineralize more readily through biodegradation^58^. Natural resources such as cellulose, starch, polylactic acid (PLA), and polyhydroxyalkanoates (PHA) are often used for the production of biodegradable plastics^58–60^. Internationally recognized standards, EN 13,432 (European), ASTM 6400 (USA) or ISO 17088 (International) are used to define and label the biodegradability of plastic materials^59,60^. According to these standards, a plastic product can be “compostable-labelled” if at least 90% (weight) disintegrates into particles that pass through a 2 × 2 mm mesh within 3 months and mineralize within 6 months in an industrial composting environment. According to these laboratory tests, plastic items are mixed with biowaste and typically exposed at temperatures in the range between 40 and 60 °C. Obviously, these tests do not represent the conditions prevailing in the marine environment, which points out the need to assess the fragmentation and biodegradation of compostable-labelled plastic materials in seawater.

Fragmentation rates of plastic litter are likely to vary widely according to environmental conditions and the plastic material grade in question. The rates will also not be constant in time, as the degradation results from a variety of independent and interdependent processes (e.g.biodegradation, hydrolysis, photooxidation, erosion, cracking, etc.) that do not proceed at the same rates and do not stay constant over time. Such rates have only been roughly estimated in outdoor exposure experiments in seawater, with rare attempts to determine loss of tensile strength or surface area^4,19,33,38^. The rates at which we can expect plastics to completely mineralize in the sea are expected to be very low and challenging to empirically measure or quantify^61^.

Quantifying weathering, fragmentation and mineralization rates of different types of plastic objects in a marine environment with existing methods is not straightforward^57^. We therefore designed a fit-for-purpose laboratory microcosm experiment to investigate biofouling and fragmentation of a variety of plastic objects within a relatively short time span. We determined growth and species composition of biofilms on the plastic items in the microcosm to observe if differences developed depending on the type of material. We hypothesized that weathering and release of small fragments result in the development of pores and crevices in the surface of plastic objects in the microcosm, and when these pores are filled with seawater this can be measured as a decrease of electrical resistance of the plastic objects^62,63^. Therefore, we tested the effectiveness of electrical resistance of plastic objects as a simple and fast indicator of plastics weathering and fragmentation. We analyzed weight loss to determine fragmentation caused by microplastics generation and/or biodegradation of each plastic type, to test our prediction that plastic products carrying ‘compostable’ labels would fragment faster than conventional thermoplastics in the microcosm over the course of a one-year experiment.

## Results

**Figure 1:**
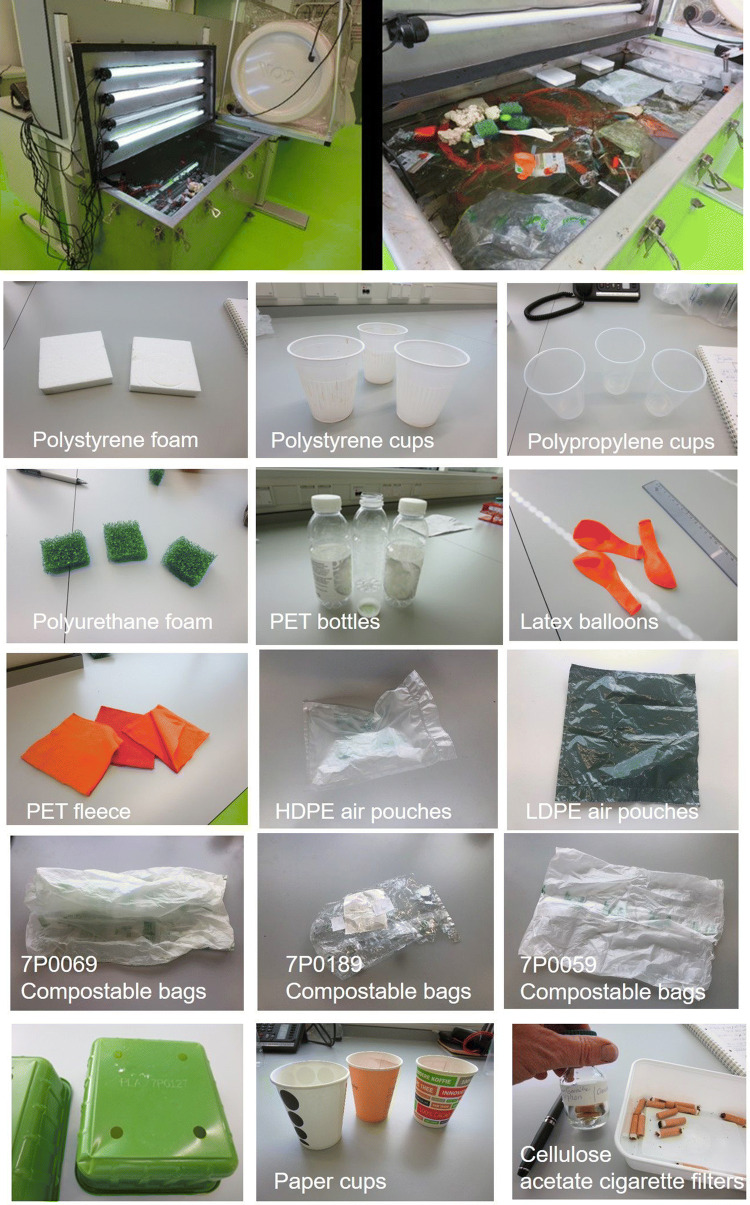
Pictures of (**A**) the marine microcosm and (**B**) added plastic objects with different polymer backbones.

Once the silicon tubes, PET bottles and fleece, PS coffee cups and the PLA materials were placed in the microcosm, they immediately sank to the bottom because of a higher density than seawater. The PU foams, cigarette filters, paper coffee cups and compostable plastic bags with registration numbers 7P0059 and 7P0069 became waterlogged and sank within 4 days. The other plastics, including the HDPE and the LDPE air pouches, latex balloons, PS foams and PP cups remained afloat on the water surface in the microcosm during the entire experiment (Fig. 1). After one year, only one of the six HDPE and two of the three LDPE air pouches had sunk to the bottom of the microcosm.

After 378–383 days in the microcosm the volume fraction (Φ) of water in different plastic items (estimated by triplicate measurements of the capacitance) were 0.79 ± 0.47 (LDPE air pouch), 1.34 ± 0.32 (HDPE air pouch), 1.38 ± 0.39 (PP cup), 1.45 (PET bottle), 2.01 ± 0.11 (PS coffee cup), and 2.18 ± 0.25 (latex balloon). This indicated the uptake of seawater into the plastic objects during incubation in the microcosm.

### Electrical resistance (ER) measurements

**Figure 2:**
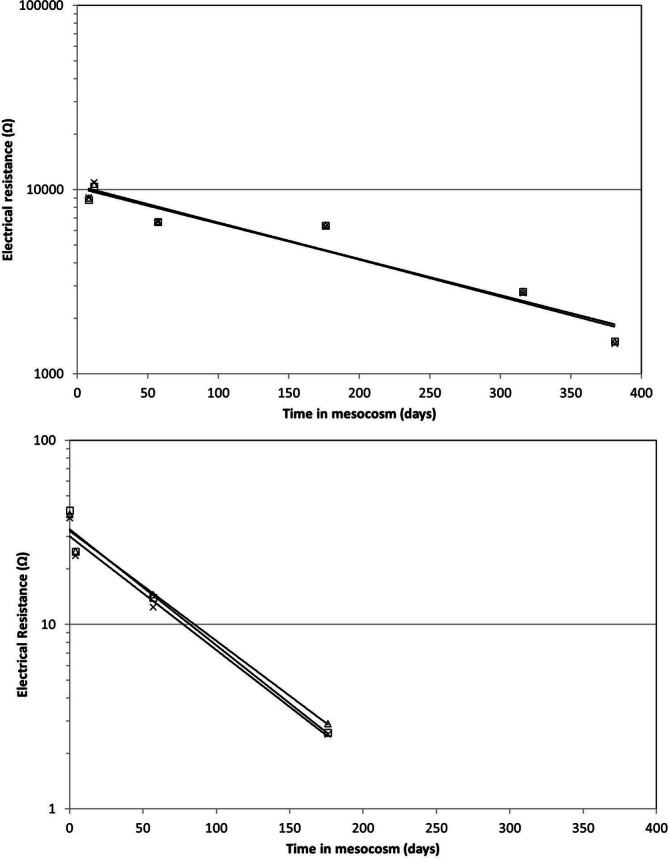
Electrical resistances of latex balloons (upper panel), and compostable trash bags 7P0069 (lower panel), measured at different times during incubation in the microcosm. Electrical resistances were recorded at 100 Hz (□), 110 Hz (∆), or 1,000 Hz (x)0 Hz (o), respectively.

The ER of different plastic materials in seawater was measured in triplicate within 8 days after incubation, and subsequently at 50 to 150-day intervals during one-year incubation in the microcosm (Table 1). Initially, ER values of the compostable-labelled plastic bags were more than two log-factors lower than those of the non-compostable plastic items. The highest ER values were found for the PET bottles. For all plastic items the ER values measured at 100 Hz, 120 Hz or 1,000 Hz decreased during incubation in the microcosm (Fig. 2). The rate constant at which the ER-values measured at 100 Hz and 120 Hz decreased ranged from 0.0045 day^−1^ to 0.0165 day^−1^, depending on the plastic material (Table 1).

**Table 1:** Initial electrical resistances (ER) of plastic objects, and ER-decrease rate constants determined during 385 days incubation in the microcosm.

Component	Material	Frequency (Hz)	Initial resistance (Ohm)	Rate constant (day^−1^)	R^2^
Air pouch	LDPE	100	13,682	0.0053	0.75
		120	13,784	0.0053	0.76
		1,000	1,989	0.0003	0.08
Air pouch	HDPE	100	6,594	0.0061	0.85
		120	6,556	0.0061	0.85
		1,000	2,483	0.0035	0.74
Coffee cup	PS	100	93,384	0.0140	0.92
		120	93,186	0.0140	0.91
		1,000	86,894	0.0138	0.91
Cup	PP	100	4,134	0.0089	0.86
		120	4,146	0.0088	0.86
		1,000	4,174	0.0088	0.86
Bottle	PET	100	183,984	0.0144	0.86
		120	187,986	0.0145	0.86
		1,000	183,993	0.0145	0.86
Bottle	PET	100	249,484	0.0165	0.90
		120	251,686	0.0165	0.90
		1,000	238,793	0.0164	0.90
Balloon	Natural Latex	100	8,794	0.0045	0.93
		120	8,976	0.0045	0.93
		1,000	9,053	0.0046	0.93
Compostable postal bag	7P0059	100	61	0.0089	0.84
		120	62	0.0097	0.84
		1,000	64	0.0075	0.96
Compostable trash bag	7P0069	100	42	0.0145	0.98
		120	40	0.0137	0.98
		1,000	38	0.0142	0.98

After 378–390 days in the microcosm, the ER values of PP and PS cups and an LDPE air pouch were measured before and immediately after removing biofilms and washing in demineralized water. We did this in order to remove seawater from the plastics. After this cleaning and washing, the ER values had increased to the range of the initial values when we started the microcosm. This indicated that low ER-values were primarily due to the uptake of seawater by the plastic items.

### Fragmentation and weight loss of the plastics

The first effects of microcosm incubation on the plastic material could be observed within the first week of the experiment. First, we observed a change in colour of the compostable trash bags with registration number 7P0069 from translucent light green into opaque white after four days of incubation. In addition, cigarette filters started to lose their paper covers.

**Figure 3:**
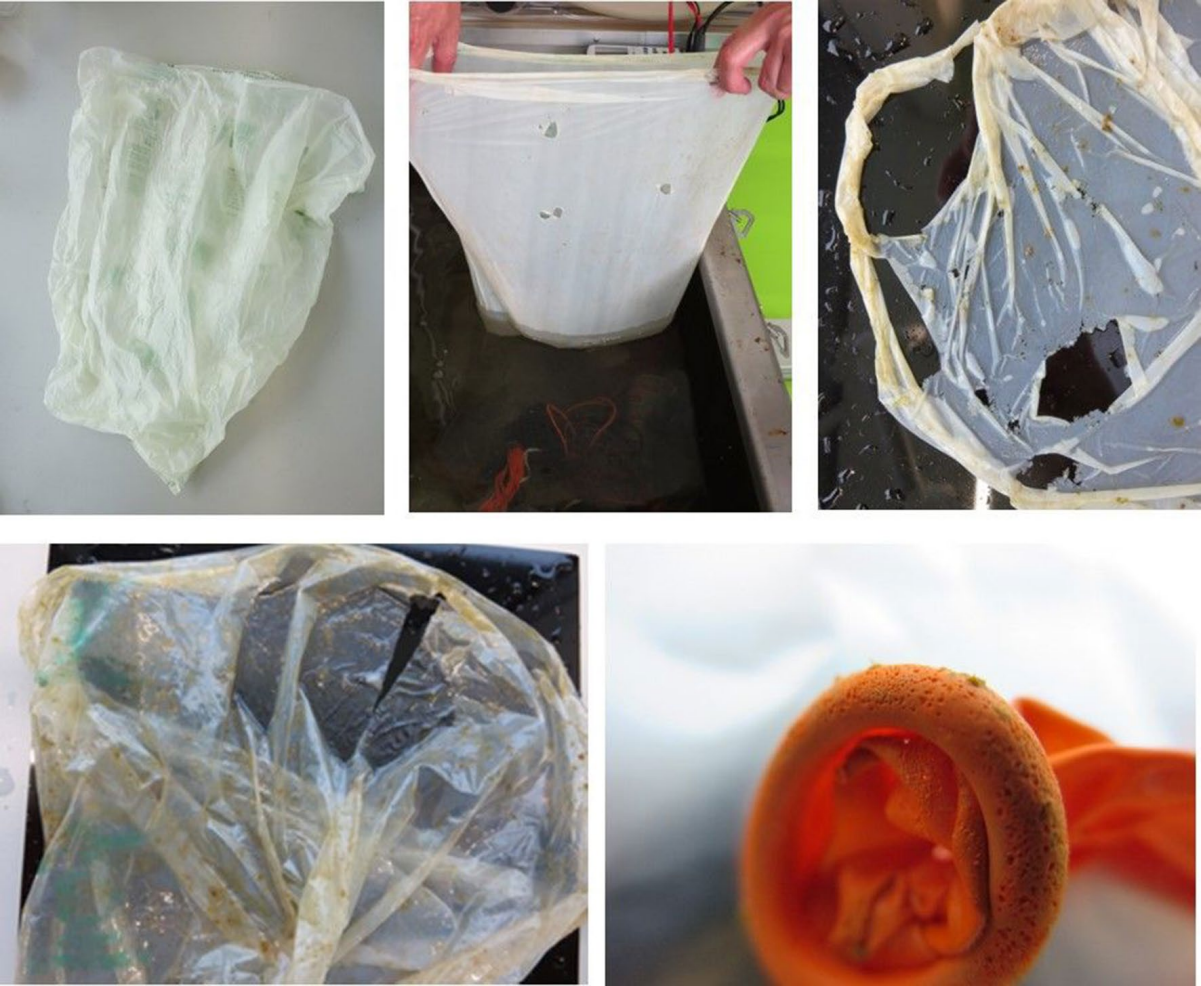
Pictures of fragmentation of compostable trash bag 7P0069 after 0, 57 and 183 days from left to right, respectively (upper panels), compostable postal bag 7P0059 after 294 days (lower left panel) and latex balloon after 383 days (lower right panel) in the microcosm.

Within two months many holes of 1 mm to 10 mm diameter appeared in the 7P0069 compostable trash bags (Fig. 3). Small particles of < 1 mm broke off of the rims of the larger holes. The compostable postal bags with registration number 7P0059 kept their integrity longer, but some holes of about 1 mm were observed after six months in the microcosm (Fig. 3).

After 378 days, loss of small crumbles (< 1 mm) was visible around the neck of the latex balloons (Fig. 3). The compostable PLA food bags and the paper coffee cups however did not show any signs of fragmentation. These materials did however become fragile and easily disintegrated upon touch. Fragmentation of the PLA bags did not result in formation of small crumbs, but rather elongated snippets. The other plastic materials showed no visible fragmentation within one year in the microcosm.

**Figure 4:**
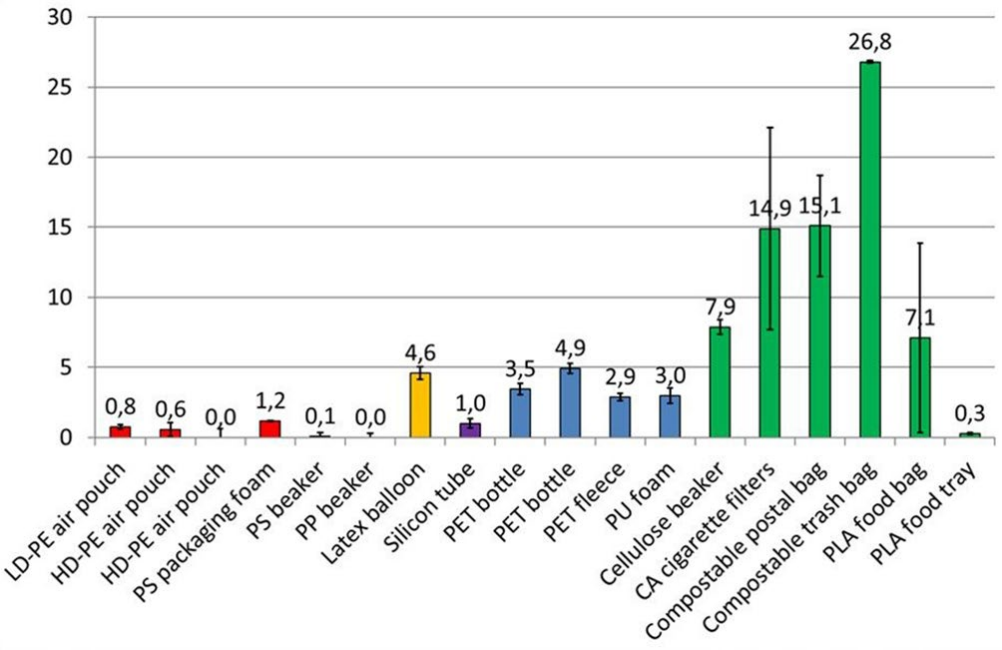
Fragmentation rates (% weight loss per year) of objects with different polymer backbones in the marine laboratory microcosm. Red bars indicate polymers with a backbone with single “C–C” carbon bonds, orange a backbone with double “C = C” bonds, purple a siloxane backbone, blue a polyester backbone, and green indicates compostable polymers.

After 378–427 days the plastics were taken from the microcosm and the dry weight of 3 to 12 replicates was determined after removing the biofilms from the surfaces (Fig. 4). The LDPE and HDPE air pouches, the PS coffee cup, the PP cup and the PLA food tray had lost less than one percent of their weight (fragmentation rates < 1% per year). Based on weight loss, the silicon tubes and the PS packaging foam had fragmentation rates of about 1% per year. Fragmentation rates of the PET materials, PUR foam and the latex balloons were between 3 and 5% per year. Higher fragmentation rates, ranging from 7 to 27% per year were found for the compostable bags and the cigarette filters. The fragmentation rate of the paper coffee cups, as natural cellulose polymer reference, was about 8% per year.

### Microplastics

**Figure 5:**
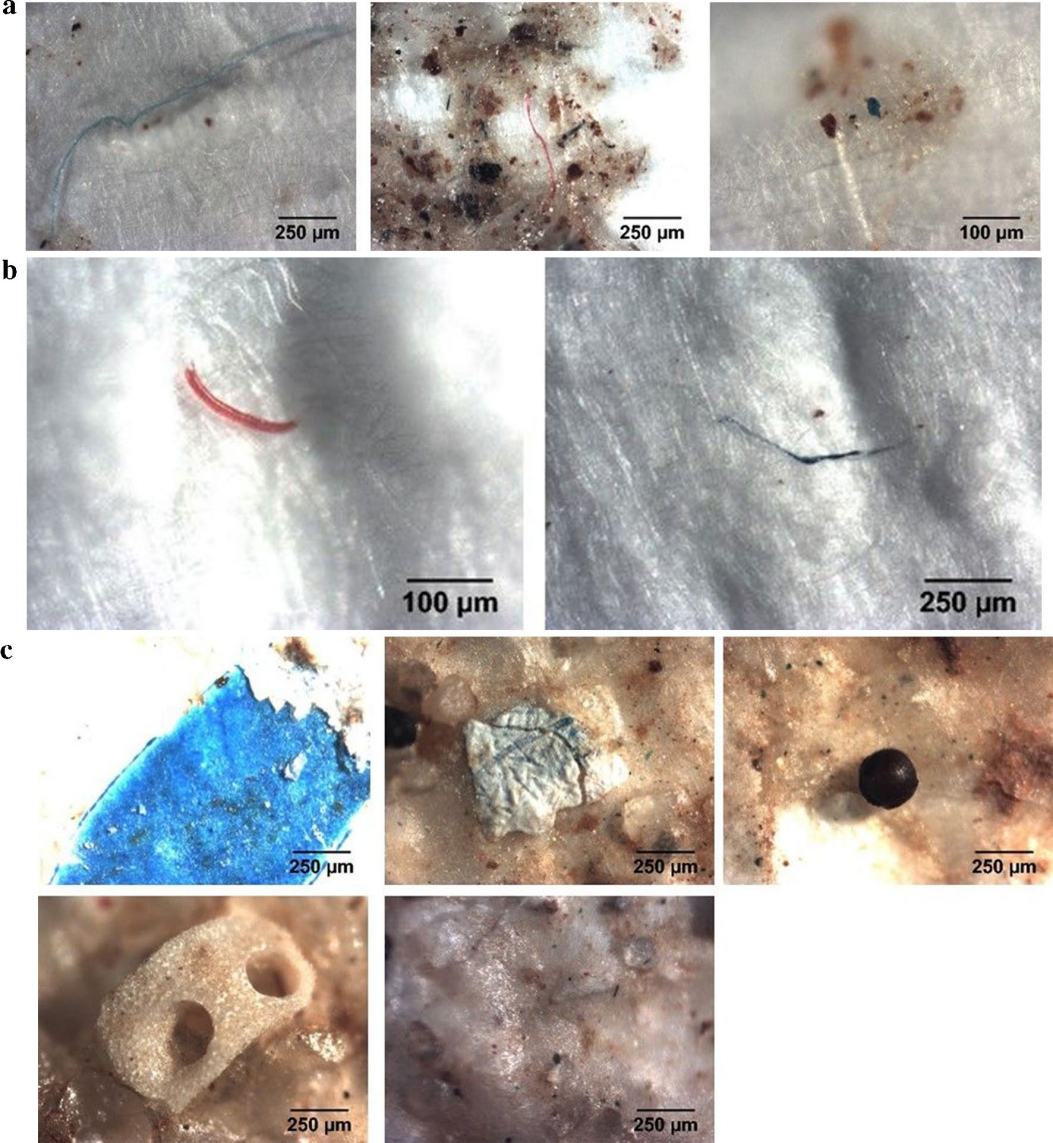
Images of microplastics and -debris from the microcosm. **a** Microdebris from the surface water, blue fiber (left), red fiber (middle), blue foil (right). **b** Red (left) and blue (right) fibers detected at 25 cm depth. **c** Microdebris from the bottom at 50 cm depth, blue foil (upper left), white foil (upper middle), brown sphere (upper right), unknown white piece (lower left), and unknown particles (lower right).

Qualitative observations with light microscopy revealed that the water samples taken from the bottom of the microcosm tank contained the highest number and variety of particles, both plastic and organic matter. Samples of water taken at 25 cm depth contained relatively low amounts of particles, mainly fibres (Fig. 5). Small plastic fragments were detected mainly in the water from the surface and on the bottom of the microcosm, indicating materials present in the plastic mixture were generally not neutrally buoyant. Numerous fibers and non-plastic debris were observed in the water samples taken from the surface and bottom of the microcosm (Fig. 5). In the reference synthetic seawater, only one particle was detected in a 522 mL sample volume, indicating a very low background level of the incubation seawater compared to the microcosm test.

### Biofouling and microbial populations on plastics

**Figure 6:**
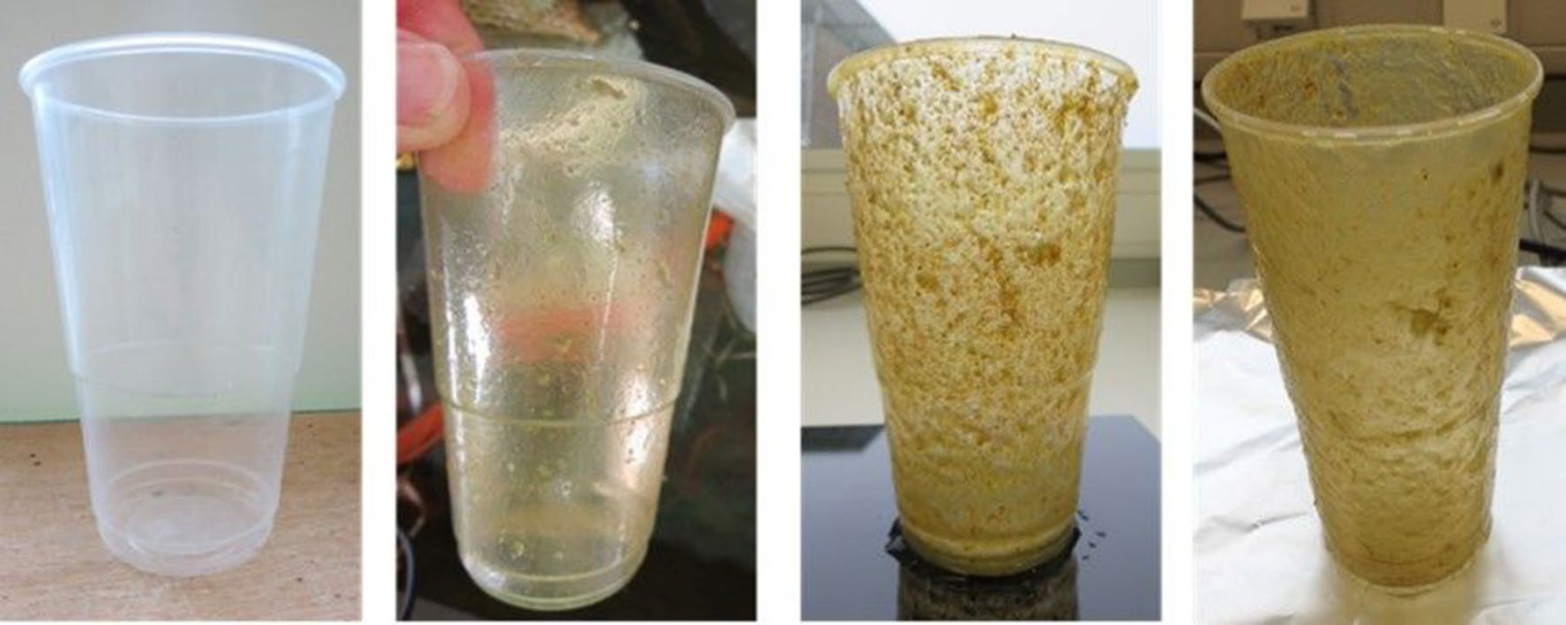
Biofouling of polypropylene cup, after (from left to right) 0, 57, 183 and 390 days in the microcosm, respectively.

Heavy biofouling occurred within several months, indicating the growth of algae, bacteria and other microorganisms on the surfaces of the plastic materials (Fig. 6). After 378–427 days in the microcosm, the wet and dry weights of the biofilms growing on 2 to 6 replicates of plastic items were determined (Table 2). Determination of the weight of biofilms growing on the cigarette filters, paper coffee cups and PLA food bags was not possible because these materials were too fragile for biofilm collection. The wet weight measurements indicated the presence of thick slimy biofilm layers on all the components added to the microcosm. The dry weight of the biofilms removed from the plastics ranged from 0.068 to 0.459 mg per cm^2^ of plastic surface exposed to the seawater, which corresponded to biofilm growth rates of 0.063 to 0.441 mg per cm^2^ per year.

**Table 2:** Quantification of biofilms on the plastics.

Component	Material	Wet weight (mg/cm^2^)	Biofilm dry weight (mg/cm^2^)	Biofilm growth rate (mg/cm^2^/year)
Air pouch	LDPE	9.6 ± 1.2	0.069 ± 0.029	0.067 ± 0.028
Air pouch	HDPE	14.4 ± 0.2	0.129 ± 0.029	0.121 ± 0.028
Air pouch	HDPE	22.2 ± 0.6	0.163 ± 0.028	0.154 ± 0.027
Tube	Silicon	24.7 ± 2.8	n.d	n.d
Packaging foam	PS	10.1 ± 7.8	0.115 ± 0.003	0.102 ± 0.003
Coffee cup	PS	16.3 ± 1.8	0.195 ± 0.088	0.186 ± 0.084
Cup	PP	39.4 ± 1.8	0.311 ± 0.095	0.291 ± 0.089
Coffee cup	Cellulose	29.5^a^	n.d	n.d
Bottle	PET	32.4 ± 2.3	0.398 ± 0.012	0.382 ± 0.011
Bottle	PET	38.7 ± 1.6	0.459 ± 0.204	0.441 ± 0.196
Fleece clothing	PET	119.9 ± 1.3^a^	n.d	n.d
Reticulated foam	PU	1.2 ± 0.9	n.d	n.d
Balloon	Natural latex	17.5 ± 2.9	0.211 ± 0.103	0.199 ± 0.098
Cigarette filters	Cellulose acetate	16.5^a^	n.d	n.d
Compostable postal bag	7P0059	13.3 ± 4.5	0.068 ± 0.038	0.063 ± 0.035
Compostable trash bag	7P0069	7.07 ± 0.04	n.d	n.d
Compostable food bag	PLA	44.4 ± 7.9	n.d	n.d
Food tray	PLA	21.3 ± 11.8	0.261 ± 0.136	0.232 ± 0.121

^a^These materials absorbed a significant amount of water.

**Figure 7:**
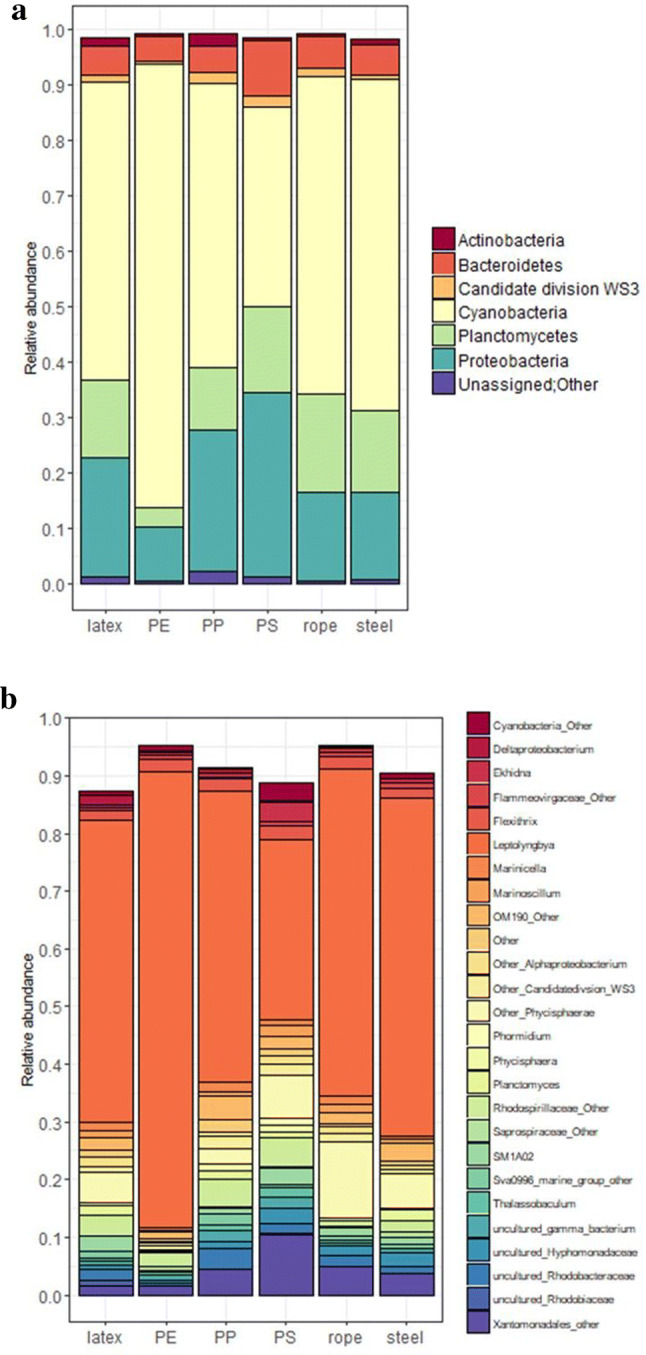
Relative abundance of 16S rRNA genes of biofilm samples from the microcosm (**A**) phylum level (**B**) genus level. Figures show only those phyla/genera, which represent at least 1% of the total community in at least one sample.

**Figure 8:**
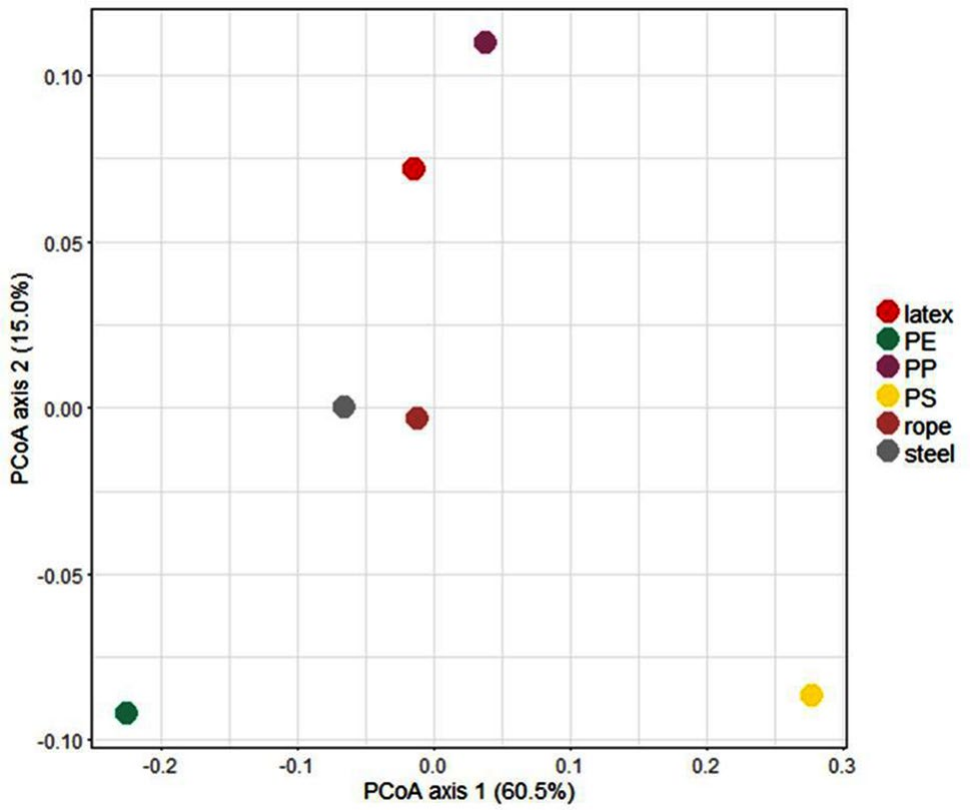
PCoA plot of the phyla and genus, indicating differences in bacterial population composition between biofilm samples from different objects.

To study the microorganisms in these biofilms, we performed a DNA metabarcoding approach on six samples, in which we focused on the bacterial community (V3-V4 16S rRNA gene sequencing). First, we checked the bacterial community diversity (Shannon–Wiener index) and the richness on the basis of the number of observed operational taxonomic units (OTUs). The lowest number of bacterial OTUs (189) was counted on the PE sample, which also corresponded to the lowest community diversity (2.02). The number of observed OTUs ranged between 307 and 452 for the other samples (Table 3). For the diversity, a mean value of 3.63 ± 0.42 was measured (Table 3). Second, we looked to the community composition on the plastics. Cyanobacteria dominated in all samples, followed by the phyla Proteobacteria, Planctomycetes and in lesser amount the Bacteriodetes (Fig. 7A). Analysis on genus level showed that one specific genus, *Leptolyngbya*, which was assigned to seven OTUs, dominated the biofilms on all plastic samples and the steel (Fig. 7B). The phyla and genus plots indicated differences in composition between samples, which are also illustrated by a principal coordinates analysis (PCoA) plot (Fig. 8). PE, PP and PS bacterial communities phylogenetically differed most from each other. In comparison, the microbial community of the rope and the steel wall showed the most resemblance.

**Table 3:** Number of operational taxonomic units (OTUs) and Shannon–Wiener diversity index obtained from bacterial 16S rRNA gene amplicons from biofilms from different materials exposed in the microcosm.

Material	Observed number of OTUs	Shannon–Wiener diversity index
Latex	452	4.15
PE	189	2.02
PP	428	4.07
PS	420	5.02
Rope	307	3.05
Biofilm from steel	390	3.49

## Discussion

Fragmentation of plastic items can be estimated with a variety of analytical techniques, based on morphological and rheological changes, or on gravimetric, scanning electron microscopy (SEM), spectroscopic or chromatographic analyses^56^. Many of these methods destroy the plastic samples during analysis and rely on the availability of expensive laboratory equipment. We therefore investigated if electrical resistance (ER) measurements can be applied as a cheap, easy to use, non-destructive alternative. A similar technique, electrochemical impedance spectrometry (EIS), is used to detect pits in paints and coatings, that are too small to be seen microscopically^63,64^. We used a simple setup to measure the ER of plastic objects submerged in seawater. The decrease of ER-values observed at low AC-frequencies (100 Hz–1,000 Hz) as a function of time is in line with EIS analyses of coatings. Capacitance measurements, and the fact that after washing in demineralized water the ER restored to initial high values, confirm that the ER measurements indicate penetration of water and ions into the plastics. One might hypothesize that this is correlated to the release of plastic particles from the macroplastic objects in the microcosm. The decrease of tensile elongation at break is a common parameter used to asses polymer degradation rates^19,33,65^. Interestingly, the degradation rate constant of 0.005 day^−1^, obtained by ER measurements in our laboratory microcosm at 24 °C for LDPE degradation, is in the range of those obtained by Andrady (1993) for different types of control LDPE sheets floating in outdoor sea experiments, using tensile elongation measurements. Degradation rate constants of LDPE sheets floating in sea at Seattle, at mean temperature of 15 °C, were 0.002–0.004 day^−1^, and at sea near Miami, with mean temperature of 29 °C, they were 0.004–0.008 day^−1^. Obviously, fragmentation rates obtained in our experiment should not be directly translated to those at sea, since in the laboratory environment temperature, light, water movement, biodiversity, chemical composition of the seawater e.g. due to metal leaching from the stainless-steel vessel are different. Our results suggest that ER-measurements may be a promising cheap and easy technique to readily monitor the initial steps of the degradation of plastics in water. Additional testing of ER-measurements, for example in freshwater and other environments is essential to asses full application possibilities of this method.

Once holes became visible in the plastic objects, ER measurements could no longer be used to measure further degradation. The holes created a direct seawater connection between the electrode inside, and the electrode outside the plastic object, with very low electrical resistance.

As an indicator of fragmentation, we determined weight loss of the plastic objects after 378–427 days in the microcosm. The observation that plastic materials composed of polymers with a carbon–carbon “C–C” backbone, PE, PS and PP, appeared most recalcitrant with a maximum fragmentation rate of 1% per year may be due to the fact that their degradation is initiated through abiotic photolytic oxidation by UV radiation^9,39^. The spectrum of the fluorescent lamps in our microcosm included UV light, but the rapid and extensive covering with microbial biofilms may have protected the plastic objects from photolytic degradation, resulting in fragmentation and measurable weight loss. In comparison, the latex balloons had fragmentation rates of about 5% per year in the marine microcosm. Natural latex is a complex coagulation of about 90% polyisoprene with carbohydrates, lipids, proteins, alkaloids, organic acids and amino acids^66^. The latex polymer consists of a backbone of isoprene monomers connected with C=C double bonds. Previous studies indicated that solar radiation as the most important environmental variable for fragmentation of latex films in freshwater and marine outdoor microcosms^67^. But in addition, latex polymers are known to be degraded by a variety of microorganisms, including marine ones^68^. Lambert et al*.* found that latex film exposed in an outdoor microcosm with artificial seawater lost weight at a rate of 50% in 87 days. The big difference to the fragmentation rates of latex material obtained in our microcosm experiment can be related to several factors: (i) a thicker membrane of our latex balloons (0.3 mm) versus the latex films (0.08 mm), (ii) exposure to a lower UV dose in the laboratory microcosm (e.g. biofouling), opposed to the outdoor microcosms, (iii) different composition or cross bonding of the latex polymers, and/or (iv) presence of plasticizers and UV absorbers in our balloons. The abundant presence of latex balloons in marine litter suggests fragmentation rates at least are not higher than rates of littering input^69^. This underpins the importance of testing degradation of real-life consumer plastic materials under realistic marine conditions. The fragmentation rates of PET and PU objects were between 3 and 5% per year. In PET and PU polymers the monomers are connected through ester bonds, which are susceptible to photo-oxidation, hydrolysis and biodegradation^9,25,27^. Hydrolysis and biodegradation may have caused further weight loss of PET and PU, than that of carbon–carbon backbone polymers. We observed the greatest weight losses for the cellulose-containing objects and the compostable plastics. Cellulose and cellulose acetate degrading microorganisms are abundant in the marine environment^70,71^. Moran et al*.* reported similar rates of weight loss of cellulosic waste in microcosms with ocean water, as we found for the cellulose coffee cups (8% per year) and the cellulose acetate cigarette filters (15% per year). Degradation of the disposable bags, labelled compostable according to the EN 13432 standard, occurred, but still 73% to 93% of their original weight remained after a year in the microcosm. Moreover, weight loss of the PLA food trays (fragmentation rate < 0.3% per year) was insignificant. The difference between weight loss of the PLA bags and the PLA food trays is most likely caused by the different PLA grades used for the production of these items^58^. This demonstrates that the current standard tests based on composting do not reflect the realistic (bio)degradation of plastic materials in the marine environment.

After a year, we detected numerous microplastic particles in the surface water and on the bottom of the microcosm. The synthetic seawater used to prepare the microcosm contained insignificant amounts of microplastics, which indicates fragmentation as a major cause for weight loss of the plastic objects in the microcosm. We did not quantify the extent to which nano- and microplastics formation, and/or mineralization, respectively, contributed to weight loss of the individual plastic items.

Biofilms on the plastics were visible within a month and increased during the experiment, indicating the presence of active growing microbial communities. The biofilm mass on the plastic materials in the microcosm (0.068–0.459 mg dry weight/cm^2^) was much lower than that on plastic objects which had been submerged for about one year in the Bay of Bengal, India (28–34 mg dry weight/cm^2^)^34,51^. Apparently, microbial growth on plastic objects in our closed microcosm system was less abundant than that on plastics exposed in the sea. The fact that one HDPE and two LDPE air pouches sunk after a year in the microcosm is in line with the observation that biofilms can increase the density of plastic objects, causing them to sink^52,72^. The impact of such ballasting on microplastics, with high surface area versus volume ratio, could be higher than on macroplastics. Recently, it was suggested that there appears to be a fast removal of plastic fragments smaller than a millimeter from the ocean surface water^11,14,73^. These experiments are congruent with the assumption that ballasting by microbial biofilms may be one explanation of this observation^19,74,75^.

Bacterial species richness was lowest in biofilms growing on a PE film and highest on the latex balloon. Possibly, the presence of many different organic biodegradable substrates in natural latex might have enhanced biodiversity on the balloon^66^. In comparison, the composition of the microbial communities growing on the plastic rope and the stainless-steel wall of the microcosm vessel showed the most resemblance. The observation that microbial populations on PS contained less phototrophs and differed most from those on the other plastics may be explained by the fact that PS items float on the water, and the sample for biofilm analysis was taken from the relatively dark underside. The biofilms on the plastic items in our microcosm appear to be significantly less diverse than those observed on plastic objects obtained from marine environments^75–78^. This may reflect the relatively simple and homogeneous setting of our artificial laboratory system. The dominance of phototrophic cyanobacteria was obviously sustained by the daily 12/12 h day/night cycle and the clear seawater, allowing the light to easily reach to the bottom of the microcosm. The fact that about 50% of the 16S rRNA genes detected corresponded to *Leptolyngbya* is significant, since this genus contains pathogenic species. This emphases the observation of other researchers, that plastic objects can act as habitats for pathogenic microorganisms^75,77,79,80^.

There is a strong need to understand what happens to plastic litter that is entering our oceans. Monitoring campaigns indicate that the amount of plastic found at the sea surface is not increasing proportionally to the estimated inputs of plastic litter and that there appears to be a short residence time of micro-meter sized particles at the sea surface^5,6,11,14,21,22,81,82^. It has been suggested that the microplastic particles in the oceans may degrade at faster rates when they become smaller, and that they continue to fragment into more hazardous nanoplastics, which may be too small to detect with current sampling techniques^19,83^. Recently it was confirmed that particles in the nanometer range are indeed formed during degradation of PS sheets^84^. We showed that a decrease in ER values may correlate to the formation of sub-microscopic pores, and thus can indicate the formation of nanoparticles. ER measurements provide thus an interesting tool to quantify the initial stage of fragmentation of plastics in seawater.

Biodegradation of compostable plastic objects in our microcosm occurred at much lower rates than in internationally recognized standard composting tests. The main reason may be that during composting tests degradation processes are routinely examined under optimized conditions that are not representative for marine environments. Our study and accumulating research indicates that even plastic materials labelled as compostable, which are meant to reduce accumulation of plastic waste, may not biodegrade and mineralize within an acceptably short timeframe in marine ecosystems^85^. Solid experimental data on long term degradation of plastic litter should therefore be collected in laboratory micro- and mesocosm systems under conditions that are realistic for the marine environment in order to best inform our understanding of marine plastic degradability.

## Materials and methods

### Setup of the marine microcosm

A stainless-steel vessel (0.6 m × 0.6 m × 1.2 m) was filled with 350 L artificial seawater (Fig. 1). The synthetic seawater was prepared by adding WesPro sea salt (www.wesdijk.nl) to demineralized water to obtain an electrical conductivity (EC) of 46 mS/cm on a WTW LF 197 EC meter (WTW Wissenschaftlich Technische Werkstätten, Weilheim, Germany). At about 10 cm below the surface, the seawater in the vessel was recirculated at 6.7 L/min with a pump (Velda Aquarius Universal 600, Groenrijk Malkenschoten, Apeldoorn, Netherlands) to create a mild constant water flow. Four fluorescent lamps (30-W, length 90 cm) were installed in the stainless-steel lid of the vessel to expose the plastics to simulated daylight, including UV-a and UV-b. The fluorescent lamps were two Zoo Med Ocean Sun T8 lamps, each generating 70 photons/m^2^/s, and two Zoomed Repti Sun 5.0 UVB lamps, each generating 60 photons/m^2^/s on the water surface of the microcosm (www.smulders.nl). Light intensities were measured with a LI-COR LI-192 underwater quantum sensor with 400–700 nm quantum response, connected to a LI-250 A light meter (CaTec b.v., Wateringen, The Netherlands). The microcosm was subjected to a 12:12 h light and dark regime. The temperature of the seawater was 24 ± 1 °C throughout the experiment. The microcosm was closed with a stain-less steel lid, to limit water evaporation and contamination with microplastics from the ambient air.

The microcosm was inoculated with 9 L seawater (conductivity 46 mS/cm) and a variety of plastic materials and stones, collected three days earlier at the North Sea beach at Katwijk aan Zee, The Netherlands. Different types of plastic items with different polymer backbones from household items were collected and added to the marine microcosm, in order to simulate a plastic contaminated marine environment (Fig. 1). The plastics included a selection of conventional thermoplastic and compostable plastic products according to the DIN EN 13,432 standard. Of each material subsamples were stored at 4 °C in the dark. A 5 cm cut was made in the packing material air pouches, to let the air escape before they were added to the microcosm.

### Electrical resistance (ER) measurements

Electrical resistance (ER) values (Ohm) of plastic materials were measured with a Voltcraft LCR 300 m (www.conrad.nl). The LCR-meter was connected to two 20 cm long messing electrodes, which were inserted inside Viton rubber tubing and fixed in a butyl rubber stopper. The electrode tips were positioned 1 cm apart from each other. At the tip of the electrodes, 2 cm messing was exposed to the seawater. For ER measurements one electrode tip was inserted in seawater inside a plastic bag, cup or bottle, respectively. The other electrode was positioned at the outside in the seawater of the microcosm. In this way, the ER of the plastics were measured by recording the resistance between the electrodes at different frequencies AC current: 100, 120 and 1,000 Hz, respectively. ER measurements at higher frequencies of 10.000 or 100.000 Hz, respectively, were not consistent and not used for this study. The ER measurements were done in the serial modus (Rs) of the LCR meter. The ER measurements were started 8 days addition after of the plastic objects to the microcosm and at 2 to 5 months’ intervals thereafter. The ER measurements were routinely recorded in triplicate, and the coefficient of variation based on 75 triplicate measurements was 25 ± 2%. The plastics ER values were calculated as follows:

$${ER}_{plastic}= {ER}_{plastic\cdot emerged\cdot in\cdot seawater}-{ER}_{seawater}$$

### Volume fraction (Φ) of water

The uptake of water by plastics was estimated by measuring the capacitance (C in nF) immediately after they were added (C start) and just before they were removed (C end) from the microcosm. The volume fraction of water of the plastics (Φ) was subsequently assessed according to the empirical relation of Brasher and Kinsbury^86^:

$$\Phi =\frac{{\mathrm{log}}\left({\mathrm{C\,end}}/{\mathrm{C\,start}}\right)}{\mathrm{log}80}$$

Values of water fractions are averages ± standard deviations of capacitance measurements obtained at 100, 120 and 1,000 Hz, respectively.

### Dry weight analyses and fragmentation rates

The weight of a selection of the plastic objects was measured before and after 378–427 days of incubation in the microcosm (Table 4). After removal from the microcosm, they were first drained for one minute before measurement of the wet weight of the plastics with the biofilms. Subsequently, the biofilms were carefully removed with a nylon brush^34^. Then the plastics were rinsed with tap water and incubated overnight in demineralized water to remove traces of seawater salts. Finally, the plastics were swept with a tissue and dried on aluminum foil in a stove at 40 °C, typically for 2 to 5 days, until constant weight. Plastics of more than 1 g were weighed on a Mettler PM 4600 balance, others on a Mettler AE 200 balance (Mettler Toledo, Tiel, Netherlands). The weight loss of 0 – 0.1% found for some PE and PP items confirmed that the biofilm removal procedure did not cause significant degradation of these plastic objects. The percentages loss of dry weight of the plastics were calculated subsequently as:

**Table 4:** Plastic objects with different polymer backbones added to the vessel, which were used for weight-loss analyses.

Component	Material	Surface area (cm^2^)^a^	Dimension (mm)	Weight before incubation (g ±SD)	n	Days in microcosm
Air pouch	Polyethylene (LDPE)	1714	210 × 204	3.330 ± 0.000	3	378
Air pouch	Polyethylene (HDPE)	1,041	205 × 127	1.087 ± 0.012	3	389
Air pouch	Polyethylene (HDPE)	1,024	200 × 128	1.450 ± 0.000	3	385
Tube	Silicon	50	100 × ɸ10	4.537 ± 0.100	2	400
Packaging foam	Polystyrene (PS)	272	100 × 100 × 18	1.495 ± 0.035	2	414
Coffee cup	Polystyrene (PS)	339	82 × top ɸ70 × bottom ɸ47	3.957 ± 0.060	3	383
Cup	Polypropylene (PP)	474	120 × top ɸ75 × bottom ɸ50	5.053 ± 0.085	3	390
Coffee cup	Paper (cellulose)	337	82 × top ɸ74 × bottom ɸ48	4.773 ± 0.015	3	418
Bottle	Polyethylene terephthalate (PET)	806	200 × ɸ65	19.240 ± 0.283	2	380
Bottle	Polyethylene terephthalate (PET)	761	220 × ɸ65	22.625 ± 0.148	2	381
Fleece clothing	Polyethylene terephthalate (PET)	900	150 × 150	9.037 ± 0.396	3	411
Reticulated foam	Polyurethane (PU)	9500^b^	50 × 50 × 19	1.297 ± 0.059	3	412
Balloon	Natural latex (about 90% cis-1,4-polyisoprene)	99	100 × 45 × 0.3	1.720 ± 0.046	3	386
Cigarette filters	Cellulose acetate in epichlorhydrin resin	6.3	25 × 8	0.253 ± 0.043	13	418
Compostable postal bag	7P0059 (blend of about 50% maize starch and 50% synthetic polyester)	2,926	331 × 221 × 0.04	3.070 ± 0.361	3	397
Compostable trash bag	7P0069 (made of compostable materials)	8,600	410 × 450	9.077 ± 0.072	3	411
Compostable food bag	7P0189 Poly lactic acid (PLA film)	1,056	230 × 165	4.258 ± 1.666	3	427
		1518	265 × 245			
		2,597	220 × 120			
Compostable food bag	7P0204 Poly lactic acid (PLA film)	1,800	225 × 200	2.300	1	427
Food tray	7P0127 Poly lactic acid (PLA)	535	125 × 115 × 42	7.975 ± 0.219	2	412

^a^This includes both the inside and the outside of the plastic surfaces exposed to the seawater.

^b^According to a volume of 47.5 cm^3^ and specific surface area of 200 m^2^/m^3^ of the foam (www.velda.com).

$$ {\mathrm{Dry}}\cdot {\mathrm{weight}}\cdot {\mathrm{loss}} \left(\%\right)=\frac{{\mathrm{W}}_{0}- {\mathrm{W}}_{\mathrm{t}}}{{\mathrm{W}}_{0}*100}$$

where ${W}_{0}$ is the initial dry weight of a plastic sample before incubation, and ${W}_{t}$ the dry weight determined after “t” days incubation in the microcosm. Fragmentation rates were subsequently expressed as % dry weight loss per year.

Biofilm removal with a toothbrush from the compostable plastic bags, labelled with registration numbers 7P0069, 7P0189 and 7P0204, respectively, was not possible without fragmenting these materials. Therefore, the biofilms from the latter plastics were removed by incubation, with 30% H_2_O_2_, overnight at 25 °C, and shaken at 100 rpm. Weight losses of triplicate subsamples of unexposed sheets of 7P0069 and 7P0189 by H_2_O_2_ treatment were 1.92 ± 1.0% and 0.094 ± 0.232%, respectively. These values were used as correction factors for dry weight loss analyses of the compostable plastic bags. Weight loss of the cigarette filters was corrected for the loss of their paper covers in the microcosm, which accounted for 49 ± 20% of their mass.

The biofilm material removed from the plastics was suspended in 100 mL autoclaved synthetic seawater and stored it at 4 °C in the dark for further analyses. The dry weight of the biofilms was determined by filtration of 25 mL subsamples of the biofilm suspensions on pre-weighed cellulose acetate filters with a diameter of 47 mm and a pore size of 0.45 µm (www.merckmillipore.com). The filters were dried in a stove at 40 °C for 3 days until constant weight.

### Microplastics

Samples were collected at 389 days after plastic addition to the microcosm. For microplastic sampling, 500 mL Erlenmeyer flasks were washed and immediately sealed with aluminum foil. Water samples (approx. 500 mL each) in triplicate were collected from the left, the middle and the right sections of the microcosm. Samples of buoyant microplastics were obtained by holding the opening of the Erlenmeyer flasks about 2 mm below the water surface. Three water samples were collected by opening the flasks 25 cm below the water surface. Samples at the bottom (50 cm depth) were withdrawn with a 100 mL glass syringe (Sanitex Eterna-Matic interchangeable) moving slowly over the bottom of the microcosm. The samples were stored at 4 °C in the dark. Microplastic particles were filtered from the water samples over a Whatman glass filter (diameter 47 mm, pore size 0.2 µm) and then rinsed with 30 mL H_2_O_2_ (30%) followed by 30 mL MilliQ® analytical grade water according to Leslie et al.^87^.

### Identification of microbial populations

Five plastic objects and one biofilm sample scraped from the steel wall with a 50 mL Greiner centrifuge tube (VWR International B.V., Amsterdam, Netherlands) were taken 665 days after starting the microcosm. The samples were kept at − 80 °C until DNA extraction and 16S rRNA gene metabarcoding^76,78^. DNA was extracted using the Powersoil DNA isolation kit (MOBIO Laboratories, Carlsbad, CA) according to the manufacturer’s instructions. The DNA extracts of all samples were stored at − 20 °C until further processing. The extracted DNA was used for bacterial (V3-V4 16S rRNA gene) taxonomic screening through amplicon sequencing using the Illumina technology (Illumina, San Diego, CA, USA). Fragments were amplified and extended with Illumina specific adaptors by using an amplification and dual-index PCR successively (detailed description in De Tender et al.). Each PCR step was followed by a PCR product clean-up using the CleanPCR reagent kit (MAGBIO, Gaithersburg, MD, USA). Quality of the final libraries was checked using the Qiaxcel Advanced with the Qiaxcel DNA High Resolution kit (QIAGEn, Germantown, MD, USA) and concentrations were measured using the quantus double-stranded DNAassay (Promega, Madison, WI, USA). The final barcoded libraries of each sample were diluted to 10 nM and equally pooled. The resulting library was sequenced on an Illumina MiSeq 2 × 300 bp paired-end by Macrogen (Seoul, South Korea), using 30% PhiX DNA as spike-in. Demultiplexing of the amplicon dataset and barcode removal was done by the sequencing provider. The raw sequence data is available in the NCBI Sequence Read Archive under the accession number PRJNA3743322. The sequence read processing was done as described in detail in^76^.

### Statistical analysis

OTU tables of the 16S V3-V4 rRNA gene amplicon sequencing were analyzed using the QIIME software package (v1.9.0)^88^. Taxonomy was assigned with the script “assign_taxonomy.py” using the uclust method considering maximum 3 database hits, with the silva v119 97% rep set (provided by QIIME) as reference for the bacterial sequences and UNITE v7 (dynamic) for fungal sequences^89–91^. For the analysis of the bacterial populations, both community diversity and composition were studied. To study community diversity, data was rarefied at 20,000 sequences. Based on this rarefied data, the number of observed OTUs and the Shannon–Wiener diversity index were calculated as an estimation of the community’s richness and diversity. Total community composition was analyzed using the multivariate analysis of the specific R package vegan (version 2.3–2)^92^. The dissimilarity matrix, based on the Bray–Curtis dissimilarity index, was calculated from the OTU table as generated by Usearch for bacterial sequences. This Bray–Curtis dissimilarity matrix was used as input for the Principal Coordinate Analysis (PCoA).

## Supplementary information

Supplementary information

## Data Availability

All sequence data of this study is available in the NCBI Sequence Read Archive under the accession number PRJNA3743322.
