# Supplementary material for: Fragmentation of plastic objects in a laboratory seawater microcosm
Source: Sci Rep. 2020 Jul 2;10:10945. doi: 10.1038/s41598-020-67927-1 (PMC7331685; doi:10.1038/s41598-020-67927-1)
Supplement: Supplementary file 1 — Supplementary information [file 41598_2020_67927_MOESM1_ESM.docx]

**Supplementary information**

**Fragmentation of Plastic Objects in a Laboratory Seawater Microcosm**

**Jan Gerritse^1*^, Heather A. Leslie^2^, Caroline A. de Tender^3,4^, Lisa I. Devriese^5^ and A. Dick Vethaak^2,6^**

**^1^ Deltares, Unit Subsurface and Groundwater Systems, Daltonlaan 600, 3584 BK Utrecht, The Netherlands**

**^2^ Department of Environment and Health, Vrije Universiteit Amsterdam, De Boelelaan 1085, 1081 HV Amsterdam, The Netherlands**

**^3^ Department of Applied mathematics, computer science and statistics, Ghent University, Krijgslaan 281 S9, 9000 Ghent, Belgium**

**^4^ Plant Sciences Unit, Flanders Research Institute for Agriculture, Fisheries and Food (ILVO), Burgemeester Van Gansberghelaan 92, 9820 Merelbeke, Belgium**

**^5^ Flanders Marine Institute (VLIZ), InnovOcean site, Wandelaarkaai 7, 8400 Ostend, Belgium**

**^6^ Deltares, Unit Marine and Coastal Systems, Boussinesqweg 1, 2629 HV, Delft, The**

**Netherlands**

**^*^Corresponding author**

**Email: jan.gerritse@deltares.nl**

**Phone: +31(0)88 335 77 75**

| Supplementary Table S1: ICP-OES analysis of elements (mg/L) in seawater used in this study | | | |
| --- | --- | --- | --- |
|  |  |  |  |
| Element | Artificial seawater - freshly prepared | Artificial seawater from microcosm - after 2092 days | North Sea water from Katwijk aan Zee (NL) |
| Al | 0,17 | 0,14 | 0,13 |
| B | 0,98 | 0,87 | 2,26 |
| Ba | 0,26 | 0,25 | 0,02 |
| Ca | 277 | 201 | 195 |
| Fe | 0,05 | 0,09 | 0,06 |
| K | 267 | 182 | 172 |
| Li | 0,27 | 0,21 | 0,27 |
| Mg | 1269 | 1127 | 560 |
| Mn | 0,004 | 0,065 | 0,005 |
| Mo | <0,02 | <0,02 | <0,02 |
| Na | 8008 | 7117 | 7985 |
| P | <0,001 | <0,001 | <0,001 |
| S | 515 | 393 | 741 |
| Si | 2,28 | 1,04 | 1,01 |
| Sr | 0,44 | 0,10 | 3,53 |
| V | <0,0001 | 0,001 | <0,0001 |
